# Oxygen – the forgotten nutrient

**DOI:** 10.1017/jns.2017.53

**Published:** 2017-09-04

**Authors:** Paul Trayhurn

**Affiliations:** 1 Clore Laboratory, University of Buckingham, Buckingham, UK; 2 Obesity Biology Unit, University of Liverpool, Liverpool, UK

**Keywords:** Hypoxia, Hypoxia-inducible factor-1, Oxygen deficiency, Oxygen utilisation, HIF, hypoxia-inducible factor, pO_2_, O_2_ tension

## Abstract

O_2_ is essential for the maintenance and growth of aerobic animals, similar to the essentiality of what are classically considered nutrients. Nevertheless, O_2_ is not customarily regarded as a nutrient, this reflecting the route by which it enters the body – through the lungs or gills in vertebrates, rather than via the mouth and gastrointestinal tract. A relative deficiency of O_2_ occurs at high altitudes and during deep-sea diving, to which distinct adaptations occur. Deficiency is also evident in lung diseases such as emphysema. Without O_2_, mitochondrial respiration and oxidative phosphorylation cannot take place. At a molecular level, cells adapt to O_2_ deficiency by switching from oxidative metabolism to anaerobic glycolysis and there are changes in the expression of a multiplicity of genes, driven by hypoxia-sensitive transcription factors, particularly hypoxia-inducible factor-1. It is argued that O_2_ should be fully included within the remit of nutritional science alongside the other essential macronutrients.

The core of nutritional science has long been the provision of macro- and micronutrients, the processes by which they are taken up by the body, the metabolic, molecular and cellular systems with which they are involved, and the consequences for the maintenance of health and the prevention of disease of either under- or overprovision. The macronutrients are customarily defined as encompassing proteins, carbohydrates and lipids, while micronutrients refer to the multiplicity of vitamins, minerals and trace elements that are required for normal physiological function. Perusal of any textbook of nutrition will show chapters devoted to each of these groups of nutrients. However, one major macronutrient that does not feature is O_2_ – other than indirectly in relation to energy expenditure and metabolic rate in the context of energy balance and substrate utilisation (respiratory quotient). Indeed, O_2_ may not even be listed in the index, reflecting the fact that many would not consider it to be a nutrient as such. This then raises the question of what is a nutrient?

The *Oxford English Dictionary* defines a nutrient as ‘a substance that provides nourishment for the maintenance of life and for growth’; other definitions include ‘any substance or matter that is needed for the life and growth of living things’ (*Webster's*). O_2_ is self-evidently an essential requirement for all aerobic organisms, and given such definitions it is unambiguously a nutrient. The explanation for why O_2_ is invariably ignored as a critical nutrient lies in the route by which it is obtained – nutrients are regarded as being delivered from the diet through the mouth and via the gastrointestinal tract. O_2_, in contrast, is procured by a distinctly different route – from the ambient air via the lungs in terrestrial vertebrates, and from the surrounding water through the gills in fish (Table 1).

**Table 1.** Comparison of the characteristics of oxygen with other nutrients

	Classical nutrient	O_2_
Source	Provided by the diet	Obtained from the ambient air (surrounding water in fish)
Route of entry	Via the mouth and gastrointestinal tract	Through the lungs
Frequency of provision	Periodic	Essentially constant
Processing	Requires processing – digestion and absorption	No prior processing required
Transportation	Transport to tissues in some cases via specific transporters	Transported directly via a specific transporter (Hb)
Storage	May be stored temporarily (e.g. glycogen in liver and muscle, lipids in adipose tissue)	Limited storage – only in muscle (myoglobin) for local use
Deficiency	Recognised deficiency diseases	Generally abundant, but relative deficiency at high altitudes, during diving and in lung disease Extensive cellular adaptations to low levels
RDA	Yes	No

This article considers O_2_ as a nutrient (macronutrient) and the similarities and dissimilarities that are evident in comparison with the recognised, classical nutrients.

## Provision and delivery of oxygen

In mammals, and other higher animals, there is a requirement for what is in effect the continuous, or virtually continuous, delivery of O_2_. This contrasts with other nutrients, the provision of which is episodic, most mammals being ‘periodic feeders though constant metabolisers’. A relative lack of O_2_ occurs in specific situations, either continuously in the case of terrestrial species living at high altitudes, or acutely and temporarily such as with aquatic animals undergoing deep-sea dives. Even some terrestrial animals living at sea level may be periodically exposed to low levels of O_2_, such as the naked mole-rat in its underground burrows^(^^1^^)^. When acute or chronic O_2_ deficiency is part of the ecological niche or environmental circumstances to which a species is customarily exposed, selective adaptations have evolved. For example, in the naked mole-rat resistance to near anoxic conditions is sustained by utilising fructose as a fuel in glycolysis, thereby bypassing the key regulatory glycolytic enzyme phosphofructokinase^(^^1^^)^.

Once taken up by the lungs, O_2_ is distributed in essence immediately and directly to tissues and cells throughout the body, needing no prior processing before being made available. In contrast, the classical nutrients normally require release from the complex structures of the foods in which they are present, as well as processing to a form that can be transferred out of the interior of the gastrointestinal tract (as with polysaccharides and TAG). In addition, in many cases once food has been digested the nutrients released are transported from the gastrointestinal tract via substrate-specific transporters (e.g. Na^+^-dependent glucose transporter and amino acid transporters) rather than moving passively across cell membranes.

Following transfer from the lungs (or gills) into the circulation the handling of O_2_ becomes more similar to that of other nutrients. O_2_ is transported to tissues and cells by the specific Fe-containing metalloprotein, Hb, located in the cytoplasm of erythrocytes in vertebrates. This has parallels with the delivery of a number of other nutrients, such as lipids and retinol, to the sites where they are required. Not all nutrients are transferred directly to the site of action – many, including glucose (as glycogen), fatty acids (as TAG), and vitamins such as retinol and vitamin D, are first stored prior to being delivered to the sites where they are required – with the liver and white adipose tissue being key storage organs. O_2_ is stored to a limited extent in muscle, bound to myoglobin, for local use only within the tissue, and this is especially evident in marine mammals that undergo apnoea when diving^(^^2^^)^.

## Metabolic functions

Despite not being considered within the remit of nutritional science in whole-body terms, at a cellular level O_2_ is recognised as a critical factor without which respiration and other key metabolic processes cannot take place. Oxidative metabolism, particularly the catabolism of fatty acids and glucose with the production of ATP through oxidative phosphorylation and mitochondrial respiration, requires a continuous supply of O_2_. Metabolic pathways central to this include glycogenolysis, glycolysis, lipolysis and the citric acid cycle, and involve cytochrome enzyme systems within the mitochondria^(^^3^^)^.

The number of mitochondria in a cell, and whether there is a highly developed cristae structure within these organelles, varies according to the extent to which each cell type undergoes oxidative metabolism and consumes O_2_. Brown adipocytes in rodents adapted to the cold, for example, have large numbers of mitochondria with densely packed cristae, reflecting the exceptionally high levels of fatty acid oxidation and O_2_ consumption needed for thermoregulatory heat production (thermogenesis)^(^^4^^)^.

## Oxygen deficiency states

The complete absence of O_2_ leads to death within minutes in man and other mammals. In addition to environmental circumstances in which a relative lack of O_2_ occurs related to the ecological niche of a species, there are certain disease states, primarily lung diseases such as pulmonary fibrosis and emphysema, where the provision of O_2_ to the body as a whole is impaired^(^^5^^)^. There are also states of cyclic O_2_ lack, as in obstructive sleep apnoea, which is one of the disorders particularly associated with obesity^(^^6^^)^. In each of these cases the overall availability of O_2_ is limited, though not necessarily to a specific tissue. O_2_ deficiency can be ameliorated, both acutely and chronically, whether in lung disorders such as chronic obstructive pulmonary disease or in medical emergencies, by increasing the provision through O_2_ therapy.

The O_2_ tension (pO_2_) of inspired air at sea level is 160 mmHg and in alveolar blood it is approximately 104 mmHg, while the general level of oxygenation in tissues is of the order of 40–50 mmHg^(^^7^^–^^9^^)^. However, some tissues have a markedly lower pO_2_, examples including the retina, thymus and spleen, with a pO_2_ of 2–25, 10 and 16 mmHg, respectively^(^^7^^–^^9^^)^.

As well as low levels of O_2_ being characteristic of certain tissues under normal circumstances, local deprivation also occurs in specific pathological situations. These include the site of wound healing, in the heart in ischaemic disease, in tumours, and in white adipose tissue depots in obesity^(^^7^^–^^10^^)^. The pO_2_ of solid tumours can be so low that those cells at the centre may be effectively anoxic. In the case of white fat, a reduced pO_2_ has been documented in white adipose tissue depots of obese rodents^(^^11^^–^^13^^)^, the pO_2_ being >3-fold lower than in lean animals^(^^11^^,^^14^^)^. Adipose tissue hypoxia in obesity is considered in part to reflect the considerable size of enlarged white adipocytes in relation to the normal diffusion distance of O_2_ in tissues^(^^9^^,^^15^^)^. This hypoxic state is linked to inflammation and fibrosis, and is considered to be a key factor underlying the changes in adipose tissue function that lead to the development of the major obesity-associated diseases, particularly insulin resistance and the metabolic syndrome^(^^9^^,^^15^^,^^16^^)^.

## Metabolic and cellular adaptations to oxygen deficiency

**Fig. 1.**
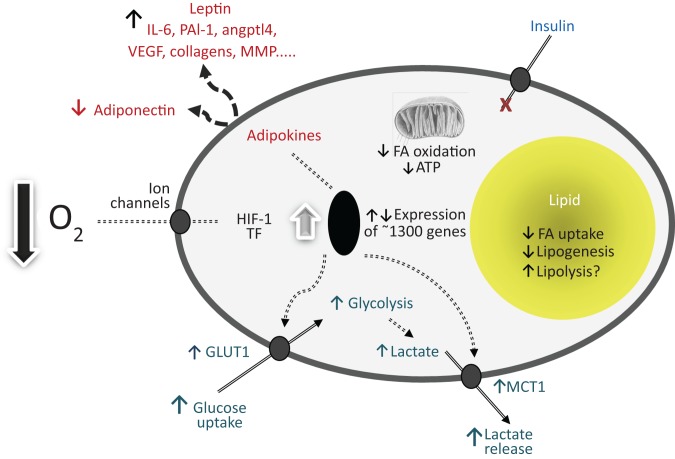
Schematic illustration of the key cellular responses to oxygen deficiency based on white adipocytes. The effect of low oxygen tension on gene expression, glucose uptake and utilisation, lipid metabolism and the production of selected adipokines is shown. PAI-1, plasminogen activator inhibitor-1; angptl4, angiopoietin-like protein-4; VEGF, vascular endothelial growth factor; MMP, matrix metalloproteinases; FA, fatty acid; HIF-1, hypoxia-inducible factor-1; TF, transcription factors (additional to HIF-1, hypoxia-inducible factor-1); GLUT1, facilitative glucose transporter 1; MCT1, monocarboxylate transporter-1.

Part of the response to a chronic deficiency of O_2_ in a tissue is the stimulation of angiogenesis in order to extend the vasculature. At the level of the cell, a local deficiency of O_2_ leads to extensive metabolic changes^(^^7^^,^^8^^,^^17^^,^^18^^)^ (Fig. 1). Glucose and lipid oxidation, oxidative phosphorylation and mitochondrial respiration fall, and there is a compensatory increase in substrate flux through anaerobic pathways^(^^7^^,^^8^^,^^18^^)^. In particular, the rate of glycolysis is greatly increased with lactate being the end product rather than pyruvate^(^^7^^,^^8^^,^^18^^)^; under aerobic conditions pyruvate is oxidised via acetyl CoA and the citric acid cycle. Elevated rates of glycolysis are driven by increases in glucose uptake through the recruitment of GLUT1, the basal facilitative transporter, and raised levels of key glycolytic enzymes^(^^7^^,^^8^^)^. Tumours have, of course, long been recognised to produce substantial quantities of lactate, reflecting their marked hypoxic state^(^^7^^,^^19^^)^. Similar observations have been made on white adipocytes maintained under hypoxic conditions^(^^9^^,^^20^^)^.

The range of metabolic changes resulting from low pO_2_ extends well beyond the augmentation of glycolysis. In the specific case of white adipose tissue, microarray studies have indicated that the expression of approximately 1300 genes is altered in adipocytes exposed to 1 % O_2_ (Fig. 1) compared with those incubated under ‘normoxic’ conditions (21 % O_2_)^(^^21^^)^. In addition to glucose utilisation, lipolysis and lipid oxidation, the pathways and functions altered in fat cells in response to low pO_2_ include cell-to-cell signalling and interaction, amino acid metabolism, and cell death^(^^21^^)^. This is reflected in changes in the amounts of encoded transporters, enzymes, and key proteins such as adipokines – including those associated with the inflammatory response^(^^9^^,^^20^^)^. Cells not only respond to major differences in pO_2_, but again, as illustrated in adipocytes, they appear to carefully titrate small variations in pO_2_ with alterations in gene expression and glucose utilisation^(^^22^^)^.

The cellular sensing of O_2_ deficiency is initiated at the cell membrane primarily through K^+^ ion channels^(^^23^^)^ and the intracellular response is transmitted by hypoxia-sensitive transcription factors which regulate the expression of hypoxia-sensitive genes^(^^7^^,^^8^^,^^17^^–^^19^^)^. The most important of these transcriptional signals are the hypoxia-inducible factors (HIF), particularly HIF-1 which is termed the ‘master regulator of O_2_ homeostasis’^(^^18^^)^. HIF-1 consists of two subunits – HIF-1β, which is constitutively expressed, and HIF-1α which is continuously synthesised and degraded but is stabilised when pO_2_ is low, this enabling the formation of the functional transcription factor^(^^8^^,^^17^^,^^18^^)^. The transcription of multiple genes is directly regulated by HIF-1, including GLUT1, glycolytic enzymes, vascular endothelial growth factor (VEGF), angiopoietin-like protein-4 and the adipocyte hormone leptin^(^^7^^–^^9^^,^^20^^)^. VEGF is, of course, a key angiogenic signal, the growth of the vasculature being central to the delivery of O_2_ as well as of other nutrients.

## Coda

O_2_ has been a forgotten, or at the very least highly neglected, nutrient. It is absolutely critical for all aerobic animals, and for most higher species is required on a continuous basis. It is essential for cellular respiration and for a host of other metabolic processes. States of deficiency are recognised and can be ameliorated. Cells have the ability to adjust to acute or chronic changes in O_2_ availability, this involving alterations in the expression of a multiplicity of hypoxia-sensitive genes regulated by key transcription factors.

Despite the similarities between O_2_ and other nutrients, there are some differences beyond the route of delivery. There is no meaningful equivalent of the RDA, and in most circumstances O_2_ is both abundant and freely available, and requires no prior processing. In contrast to many other nutrients, excess is difficult to achieve though toxicity is evident in artificially induced hyperoxaemia. It is argued that O_2_ should be viewed as firmly residing within the purview of nutritional science.
